# It takes two to tango: How immune responses and metabolic changes jointly shape cardiac Chagas disease

**DOI:** 10.1371/journal.ppat.1011399

**Published:** 2023-06-01

**Authors:** Azadeh Nasuhidehnavi, Laura-Isobel McCall

**Affiliations:** 1 Department of Chemistry and Biochemistry, University of Oklahoma, Norman, Oklahoma, United States of America; 2 Department of Microbiology and Plant Biology, University of Oklahoma, Norman, Oklahoma, United States of America; 3 Laboratories of Molecular Anthropology and Microbiome Research, University of Oklahoma, Norman, Oklahoma, United States of America; University of Wisconsin Medical School, UNITED STATES

In the last decade, the field of immunometabolism has made rapid advances and had an emerging impact in the field of microbial pathogenesis, including parasitic diseases. Chagas disease (CD) caused by the protozoan parasite, *Trypanosoma cruzi (T*. *cruzi)* is associated with the alteration of both metabolic and immune pathways in the host. Following infection, the acute phase of CD develops with nonspecific symptoms such as fever and fatigue. If untreated, after the acute phase, infected individuals enter the chronic (indeterminate) phase, and most of them show no clinical symptoms. However, around 30% to 40% of chronically infected patients develop symptomatic CD with chronic chagasic cardiomyopathy (CCC) as the most damaging clinical manifestation [1]. Recent research has shown that CCC is associated with heart metabolic changes, which can be influenced by both the immune response and parasite manipulation. Here, we discuss the main immunometabolic changes occurring during acute and chronic cardiac CD and highlight emerging future research focus areas.

## Innate and adaptive immune responses during acute and chronic Chagas disease

Both the innate and adaptive immune responses act together to control *T*. *cruzi* infection. However, the parasite can manipulate host effector immune mechanisms and evade antiparasitic activities, resulting in a lifelong infection. Antigen-presenting cells, including macrophages and dendritic cells, represent a first line of defense that encounter *T*. *cruzi* and recognize parasite-derived antigens through pattern recognition receptors, including Toll-like receptors (TLRs). Induction of TLR-dependent signaling pathways leads to activation of myeloid differentiation primary response protein 88 (MyD88) and nuclear factor-κB (NF-κB), and production of pro-inflammatory cytokines including interleukin (IL)-12 and tumor necrosis factor (TNF)-α. IL-12 induces production of interferon-γ (IFN-γ) in natural killer cells as well as adaptive T helper 1 (Th1) cells. During acute infection, IFN-γ is considered the main inflammatory cytokine in restricting *T*. *cruzi* growth by induction of nitric oxide (NO) in macrophages and up-regulation of adhesion molecules and chemokines resulting in leukocyte infiltration into cardiac tissue (for more details see, [1]). However, an increased population of infiltrating leukocytes producing IFN-γ and TNF-α has been associated with cardiac damage in chronic CD patients. Thus, IFN-γ production can result in distinct outcomes depending on CD stage. On the other hand, production of immune-regulatory cytokines such as IL-10 lessen the deleterious effects of pro-inflammatory responses [1]. Additionally, Th17 cells are associated with a protective response during acute CD through IL-21–dependent increased proliferation and activation of CD8^+^ T cells, leading to decreased parasitemia and improved survival [2]. Importantly, a balanced state between pro- and anti-inflammatory immune responses is associated with asymptomatic chronic CD, whereas an excessive pro-inflammatory response can result in cardiac pathology [3].

## Metabolic profile and impact on immune responses in cardiac tissue during *T*. *cruzi* infection

In acute *T*. *cruzi* infection in mice, the demand for glucose increases in the heart, as evidenced by elevated cardiac glucose and increased glycolytic metabolites such as glucose-6-phosphaste, fructose-6-phosphate, pyruvate, and lactate [4]. Also, in vitro *T*. *cruzi* infection of cardiomyocytes up-regulates glycolytic intermediates and enzymes [5]. This phenomenon could stem from the high energy demand of amastigotes for replication [6]. Acute *T*. *cruzi* infection causes a decrease in the cardiac tricarboxylic acid (TCA) cycle metabolite fumarate [4]. Interestingly, an unbalanced TCA cycle appears in in vitro *T*. *cruzi*–infected cardiomyocytes as shown by increased succinate that was not followed by elevated downstream metabolites such as malate and fumarate [5]. It should, however, be noted that in vitro systems may not fully mirror in vivo infection, since they present with high multiplicity of infection, nutrient-rich medium with optimized gas exchange conditions, usually only one host cell type, and can only cover short (acute-like) time points.

Mitochondrial oxidative phosphorylation was also decreased in the heart of chronically *T*. *cruzi*–infected mice [7]. In contrast, oxygen consumption rate is increased during in vitro infection of cardiomyocytes [5]. Long chain fatty acids and long chain acylcarnitines increased in acute CD [4], with the balance between shorter versus longer chain acylcarnitines and glycerophosphocholines favoring long chain lipids in nonfatal acute infection outcomes [8]. In contrast, the level of most cardiac acylcarnitines decreased in one chronic *T*. *cruzi* infection model [9]. Th1-activated macrophages metabolize arginine through inducible nitric oxide synthase (iNOS) and generate NO, leading to destruction of intracellular *T*. *cruzi* [10]. The pentose phosphate pathway (PPP) is crucial for NO production in *T*. *cruzi*–infected macrophages stimulated with IFN-γ [11]. On the other hand, *T*. *cruzi*–derived proteins such as cruzipain can induce an alternate type 2 immune response, activation of arginase-1, L-arginine catabolism, and, subsequently, production of polyamines, which enhance parasite proliferation in macrophages [12]. During acute CD in mice, there is a decrease in the levels of L-arginine and citrulline (an iNOS activity indicator) in heart tissue, while the polyamine putrescine increases, suggesting activation of a dominant polyamine pathway over NO production and parasite clearance [10]. Treatment of *T*. *cruzi*–infected mice with L-arginine results in increased NO production and reduced cardiac parasite burden, leading to heart function improvement, increased survival and improved clinical score [10]. Thus, targeting L-arginine metabolism in CD may provide additional treatment strategies.

## The possible roles of immune responses in reshaping tissue metabolite profiles during *T*. *cruzi* infection

Following intracellular pathogen infection, inflammatory cytokines can modify cellular metabolism in distinct tissues. For example, in viral infections, IFN-γ induces down-regulation of insulin receptors on skeletal muscle, leading to insulin resistance and glucose metabolism dysregulation [13]. Interestingly, up-regulation of IFN-α and IFN-γ signaling pathways in *T*. *cruzi*–infected cardiomyocytes is positively correlated with increased expression of hypoxia and glycolysis genes [5]. Additionally, infiltration of leukocytes into cardiac tissue may also contribute to the observed increase in glycolysis, as glycolysis fuels antiparasitic responses in activated immune cells and is essential for restriction of the related parasite, *Leishmania donovani*, in mice [14]. Mechanistically, glycolytic activation by hypoxia-inducible factor-1α, an important inducer of many immune functions, leads to increased *T*. *cruzi* infectivity and replication [5]. IFN-γ and TNF-α can directly cause mitochondrial dysfunction, affecting fatty acid oxidation [15]. Type I interferons (IFN-I) may also be involved in the metabolic perturbations during CD. In acute experimental CD, deficiency in IFN-I receptor causes disease tolerance without affecting tissue parasitic burden [16]. The lack of antiparasitic function for IFN-I signaling may reflect a metabolism-modulating function, as in vitro treatment with IFN-I cytokines can increase glycolysis in adipocytes [17], and activate the tryptophan-kynurenine pathway in hepatocytes [18]. Therefore, inflammatory mediators may induce certain metabolite changes influencing disease outcome. However, the in vivo effects of inflammatory cytokines on tissue metabolites during CD are still undefined.

Although immune responses play essential roles in parasite elimination and maintenance of tissue homeostasis during CD, excessive exposure to inflammatory mediators can contribute to histopathologic outcomes. This matter has been confirmed for the most destructive sequela of chronic CD, heart fibrosis, which is associated with inflammatory cell infiltration, cardiac fibroblast activation, and fibroblast to myofibroblast differentiation. Previously, we have shown that cardiac fibrosis is significantly correlated with heart metabolic alterations, and serum levels of profibrotic cytokines are also significantly correlated with heart metabolic alterations in chronic CD in mice [19]. Up-regulation of fibrogenic cytokines such as tumor growth factor (TGF) is one of the factors influencing cardiac fibrosis in chronic CD. TGF-β treatment of primary lung fibroblasts instigates metabolic changes such as increased glycolysis, as evidenced by increased lactate production. Up-regulation of glycolytic enzymes is required for myofibroblast differentiation and collagen production [20]. Thus, TGF-β induction may be one of the causes of the observed increased glycolysis in CD [4]. Metabolic dysregulation induced by immune responses offers key mechanisms underlying heart fibrosis during chronic CD.

## Interrelated metabolic and immune response changes following Chagas disease treatment

Currently, nifurtimox and benznidazole (BZ) are the treatments of choice for CD. Both drugs are unable to completely restore the host metabolic profile. Nifurtimox treatment restores most serum fatty acid levels as well as glutamine and taurine in chronic CD patients. However, nifurtimox does not renormalize the total serum metabolic profile [21]. Similarly, we have shown that BZ treatment alone significantly decreases parasite load in all heart regions but does not fully recover immune responses, cardiac metabolome, and electrical functions in chronic CD in mice even after 56 days posttreatment [22]. In addition, BZ administration in the chronic stage leads to parasite clearance, but only partial changes in the immunological responses in chronic CD patients [23]. Therefore, perturbed metabolome and immunity could be factors that continue to drive CCC pathogenesis following late-stage antiparasitic treatment. In vitro BZ treatment does not renormalize metabolic signatures of *T*. *cruzi*–infected myocytes compared to uninfected controls [24]. In addition, BZ causes metabolic perturbation in uninfected cells such as reduction of pyruvate levels [24], suggesting adverse effects of BZ on the myocyte metabolome. Interestingly, when BZ treatment is combined with a *T*. *cruzi* vaccine containing a TLR4 agonist adjuvant, cardiac metabolic profile and Th1 immunological responses are improved, despite lower parasite clearance [22]. Altogether, it is likely that immunological factors have a direct impact on metabolic alterations in the cardiac tissue. Thus, host-directed therapies targeting persistent metabolic alterations or promoting balanced immunity or immune normalization need to be considered as an adjunct to antiparasitics to improve patient outcomes.

## Future directions

**Fig 1:**
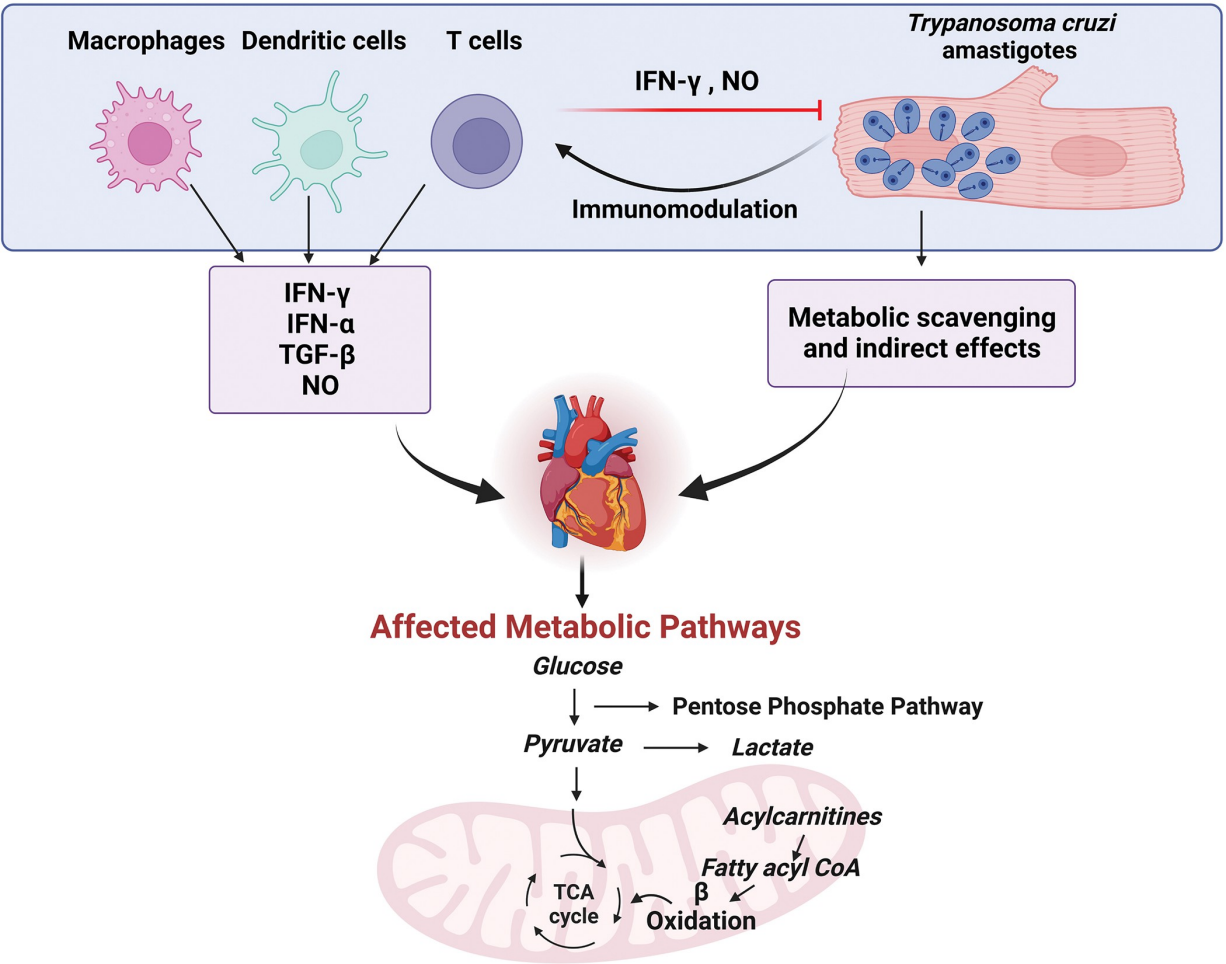
Cross-regulation of cardiac metabolic pathways by immune cell responses and *Trypanosoma cruzi* in the heart. Production of inflammatory mediators including IFN-γ, IFN-α, TGF-β, and NO can affect the metabolic profile of cardiac tissue, as can *T*. *cruzi* itself. Parasite mediators of cardiac metabolic modulation are still to be discovered. Created with BioRender.com. IFN-α, interferon-α; IFN-γ, interferon-γ; NO, nitric oxide; TCA, tricarboxylic acid; TGF-β, transforming growth factor-β.

As discussed here, there is cross-regulation of immunity and metabolism during CD (Fig 1). Given the differences in immune functions between acute and chronic CD, a more complete understanding of the metabolic pathways altered by the immune system in vivo may enable us to design more effective future therapies that can also address bystander metabolic effects of infection [25]. Specific metabolic pathways also show greater or poorer resilience following treatment [22], and metabolic modulation shows promise for CD treatment. However, these prior metabolic studies did not provide information on dynamic metabolic fluxes. Hence, metabolic flux analyses are needed for a comprehensive understanding of metabolic changes in CD. In sum, much remains to be done in future investigations to fully define the intersection between immune responses, metabolism, CD pathogenesis, and CD treatment.
